# New Assessment Tools for Risk Drinking During Pregnancy: T-ACE, TWEAK, and Others

**Published:** 1994

**Authors:** Marcia Russell

**Affiliations:** Marcia Russell, Ph.D., is a research scientist at the Research Institute on Addictions, Buffalo, New York

## Abstract

Effective screening of pregnant women for drinking that puts the fetus at risk for developing fetal alcohol syndrome is an essential part of prenatal care. Screening tools such as questionnaires have demonstrated varying degrees of accuracy in identifying risk drinkers among pregnant women.

Although much has been done to educate the public about the dangers of drinking during pregnancy, not everyone has received the message that such behavior puts the fetus at risk for acquiring alcohol-related birth defects. Routine assessments of their alcohol intake give women opportunities to ask questions about drinking during pregnancy and give health care providers opportunities to advise patients whose alcohol use may put their pregnancy outcomes at risk. Even when women have heard public health messages about alcohol-related birth defects, having their health care provider ask about their drinking turns a general message into a specific and personal one, which is more likely to evoke a positive response (Minor and Van Dort 1982). Accurate information about the risk involved and advice about drinking during pregnancy are sufficient to eliminate hazardous drinking in many or perhaps even most pregnant patients (Rosett et al. 1983). However, followup and referral to alcoholism treatment professionals are necessary for heavy-drinking pregnant women who are unable to reduce their alcohol intake. Effective linkage with such professionals is a critical component of any screening program.

It also is useful to assess retrospectively a mother’s drinking during pregnancy when making a diagnosis in a child who has developmental deficits. Documenting maternal alcohol abuse during pregnancy can help establish a diagnosis of fetal alcohol syndrome (FAS) or fetal alcohol effects (FAE). Although some health practitioners have questioned the utility of diagnosing alcohol-related birth defects because there is no known cure, such a diagnosis may help to identify why a child has problems learning or displays abnormal behavior. Recent studies indicate that some children exposed to alcohol prenatally have serious behavioral and learning deficits even though their IQ’s are normal or borderline (Streissguth et al. 1991). Because the etiology of their problems is not recognized, these children are often thought to be willfully inattentive and disobedient. Societal response is censorious, rather than constructive. Therefore, appropriately diagnosing alcohol-related birth defects in these children can be a great relief to parents and teachers and can serve as a basis for implementing remedial programs to minimize the negative consequences of the developmental deficits.

Several tools exist for assessing a pregnant woman’s drinking habits, including tests for biochemical indications of alcohol-related damage to body systems and screening procedures based on questionnaires about drinking behaviors. Of the two, questionnaires currently are the tools most frequently used in prenatal clinics. Screening refers to the mass administration of a test for a health problem to people who have not already been identified as being at risk for that problem.

Routine assessment of alcohol intake during pregnancy can be thought of as screening for risk drinking during pregnancy. Some screens for drinking during pregnancy are designed to identify levels of drinking that are unlikely to be harmful to the woman herself but that could harm the fetus. Other screens target levels associated with alcohol abuse or dependence,^1^ assuming that women who are alcoholics are risk drinkers. The purpose of screening is to identify health problems or risks in time for intervention to prevent serious consequences such as FAS. This article reviews currently used strategies for assessing risk drinking, focusing on the effectiveness of specific screening procedures.

## Evaluating Screening Methods

Screening methods usually are inexpensive and require little time to administer. In contrast, diagnostic procedures, which are designed to determine a patient’s actual condition or disorder, are often time consuming, invasive, and costly. However, because they are more accurate, diagnostic procedures are usually used to evaluate the effectiveness of screening methods. In some cases, a well-validated screening method may be used to evaluate a new screening method.

Table 1 summarizes some results of screening for risk drinking during pregnancy (see sidebar for a discussion of risk drinking). Measures that are useful in evaluating the performances of screening procedures are derived from data in such tables: sensitivity, which is the probability, or likelihood, that a woman who is a risk drinker tests positive; specificity, which is the probability that a woman who is a nonrisk drinker tests negative; positive predictive value, which is the probability that a woman who tests positive is a risk drinker; and efficiency, which is the overall percentage of women correctly identified (Hennekens and Buring 1987).

**Risk Drinking During Pregnancy**

Risk drinking during pregnancy is defined as the amount of maternal drinking associated with harm to the fetus. This amount may vary from study to study, and it may change as additional dose-response data on alcohol effects on the fetus during pregnancy become available. For example, Sokol and colleagues (1989) defined pregnancy risk drinking as an average of two or more drinks per day near the time of conception.

A recent study of prenatal alcohol exposure indicated that the threshold for clinically significant developmental deficits is an average of more than one drink a day (Jacobson et al. 1993). However, it was noted that few women in the study drank daily; therefore, average intake did not represent the typical dose. Rather, among infants born to women who drank more than an average of one drink a day, the median amount of alcohol the fetuses had been exposed to was six drinks per occasion (Jacobson et al. 1993).

—*Marcia Russell*

Sensitivity measures the extent to which the screening procedure is successful in identifying all the risk drinkers in a population, and positive predictive value measures the number of women that must be followed up with diagnostic procedures to identify one true risk drinker. For example, a positive predictive value of 20 percent indicates that diagnostic procedures will verify risk drinking in one of five patients testing positive on a screen.

It should be noted that positive predictive values and efficiency scores are influenced by the prevalence of the condition for which one is screening. As illustrated in the table, when the prevalence of risk drinking during pregnancy in a population is 5 percent, positive predictive values are low, even though sensitivity and specificity rates are fairly high. This is true because, when the prevalence of risk drinking is low, the number of true positives (i.e., the number of actual risk drinkers who tested positive on the screening test) is small in comparison with the number of false positives (i.e., the number of women not at risk who tested positive on the screening test).

A screening procedure can still be efficient in a population with a 5-percent prevalence rate even if sensitivity is low, as long as specificity is reasonably high. This can occur because failing to identify women who are true risk drinkers will not influence the number of women correctly identified by screening when there are relatively few risk drinkers in the population being screened. For example, 95 percent of the population in table 1 could be correctly identified simply by assuming there were no risk drinkers, an assumption that would yield a sensitivity of 0 percent and a specificity of 100 percent.

### The Ideal Screening Test

The ideal screening test would be both highly sensitive and highly specific, but in actuality, there is usually a tradeoff between sensitivity and specificity for any given test. Most tests yield a range of scores, with some clearly not at risk for the problem in question, others clearly at risk, and the remainder somewhere in between. In such situations, the cutoff point used to define a positive screening score is arbitrary. For example, if risk drinking was assessed by 10 questions, each one of which scored 1 point for a positive answer, women with a score of 0 would clearly not be at risk. Those with a score of 10 would clearly be at risk. “Cut point” refers to the score required to establish a positive screen. A low cut point (i.e., less than 5) would represent a lenient criterion, whereas a high cut point (i.e., more than 5) would represent a stringent criterion. If a lenient criterion is used, more risk drinkers will be identified (high sensitivity, low false negative rates), but more nonrisk drinkers also will screen positive (low specificity, high false positive rates).

Conversely, employing a stringent screening criterion will reduce the number of nonrisk drinkers who screen positive (high specificity, low false positive rate), but more risk drinkers will be missed by the screening test (low sensitivity, high false negative rates). Usually, screeners give priority to sensitivity, and, given the importance of identifying risk drinkers, this should be done whenever possible. However, specificity becomes more critical in situations in which resources are not available to follow up adequately all patients who screen positive.

## Overview of Assessment Tools

### Laboratory Tests To Screen for Risk Drinking

Laboratory tests can detect abnormalities in body biochemistry that have been caused by heavy drinking. The use of these tests to screen for risk drinking is based on the assumption that an alcohol intake heavy enough to alter a woman’s biochemistry is also heavy enough to harm her fetus. For example, alcohol-related liver damage may be signaled by elevated blood levels of enzymes such as gamma-glutamyl transferase (GGT) that are released by the injured liver cells. Heavy drinking also may cause red blood cells to swell, producing an increase in mean corpuscular volume (MCV), the average volume of a blood corpuscle. Although they are associated with alcohol abuse, these physiological changes tend to be neither sensitive nor specific to alcohol abuse (Chan 1991).

Accordingly, the laboratory tests currently available are frequently negative for risk drinking in young heavy drinkers who are otherwise in good health, resulting in false negatives, and positive in people who may not drink heavily but have a condition that produces physiological changes similar to those caused by alcohol, resulting in false positives. Because abnormal body biochemistries tend to be associated with the late stages of problem drinking, heavy drinking among women in their childbearing years is less likely to be identified by laboratory tests than by much simpler and less costly questionnaire methods.

However, if laboratory tests are available and abnormalities suggest liver damage or anemia, it would be appropriate to follow up with questions on alcohol use. This approach may lead to the identification of risk drinking in cases where it was not suspected and may assist in breaking down denial of heavy drinking. It has been demonstrated that elevated MCV and GGT values predicted levels of drinking and alcohol-related birth defects in problem drinking obstetric patients (Ylikorkala et al. 1987).

### Brief Questionnaires

Currently, brief questionnaires represent the most effective method of screening for risk drinking during pregnancy. Three basic approaches to questionnaire design exist: asking about consequences of heavy drinking, asking about alcohol intake, or asking about both. Consequences of heavy drinking include alcohol problems such as social, legal, or financial problems (e.g., loss of friends, arrest for driving while intoxicated, and loss of job), or emotion-focused problems such as feeling guilty about one’s drinking or feeling that one ought to cut down. Asking about these problems is an indirect way of identifying women who may be risk drinkers; presumably, these women would not have alcohol problems if they did not drink fairly heavily.

Asking about alcohol intake, however, is the most direct way of assessing drinking during pregnancy. In the Ten-Question Drinking History (Rosett et al. 1981), questions are asked separately for wine, beer, and liquor regarding the frequency with which each beverage is consumed during 1 week, the quantity consumed at one time, and whether the person ever drinks more than the amount reported. A final question asks whether drinking patterns have changed in the past year.

Methods such as this have several advantages. This direct, nonjudgmental approach often elicits reports of alcohol intakes high enough to indicate risk drinking and can alert health care providers to the need for intervention. The questions also serve as a direct introduction to counseling about risk drinking during pregnancy, whereas with women who report alcohol problems, followup questions on alcohol intake must be asked to obtain this information. Also, risk drinking can occur in the absence of any alcohol problems. Thus, direct questions on alcohol intake may identify risk drinkers who would be missed by indirect questions on consequences of heavy drinking.

#### MAST and CAGE

Proponents of screening indirectly for risk drinking or of asking questions about alcohol problems have been concerned that direct questions about alcohol intake may trigger denial or minimization of the amount consumed, resulting in high false negative rates. They hypothesize that denial may be circumvented by indirect questions about consequences of heavy drinking, such as alcohol problems or tolerance to the effects of alcohol.

Alcoholism^2^ screening instruments such as the Michigan Alcoholism Screening Test (MAST) (Selzer 1971) and the CAGE test (Ewing 1984), though not designed specifically for screening pregnant women, have served as sources of items for questionnaires that screen for risk drinking during pregnancy.

The MAST consists of 25 questions, many used in earlier alcoholism surveys. It was intended to provide a quantitative, structured interview for the detection of alcoholism that could be rapidly administered by professionals trained in the detection of alcoholism as well as by nonprofessional personnel.

A criticism of the MAST particularly relevant to its use with pregnant women is that it may be less sensitive to alcohol-related problems among women because it was developed and tested primarily in male populations. Another criticism is that the MAST contains many items that are directed toward the identification of severe, chronic alcoholics who recognize that they have a problem and have already sought help. Questions about symptoms of alcoholism more typical of men, such as fist fighting after drinking and arrests for driving while intoxicated, may offend women. And, compared with more recently developed questionnaires, the MAST is lengthy and difficult to score, which makes it impractical for clinical use.

The CAGE test is much shorter than the MAST, employing four clinical interview questions: (1) Have you ever felt you ought to Cut Down on your drinking? (2) Have people Annoyed you by criticizing your drinking? (3) Have you ever felt bad or Guilty about your drinking? (4) Have you ever had a drink first thing in the morning to steady your nerves or get rid of a hangover (Eyeopener)?

Studies in prenatal clinics indicate that the CAGE test is less sensitive there than when used in psychiatric settings, perhaps because women in psychiatric settings tend to be more severely alcoholic than those in prenatal clinics (Waterson and Murray-Lyon 1988). Concern also has been expressed that pregnant women may report feeling bad or guilty about having consumed any alcohol, which has the potential to reduce specificity of the CAGE test. That the CAGE test assesses lifetime, rather than current, alcohol-related problems also may reduce its specificity to risk drinking during pregnancy.

As mentioned earlier, a limitation of alcoholism-screening instruments such as the MAST and the CAGE test is that they do not identify heavy drinkers who have not experienced alcohol-related problems. However, tolerance to alcohol effects tends to develop fairly rapidly in most drinkers, whether or not they experience any alcohol-related problems. Sokol and colleagues (1989) have employed this phenomenon in their research on risk drinking in pregnancy. All of their screening studies to be discussed here have been conducted among black women attending an inner-city prenatal clinic in Detroit who reported ever consuming alcohol. Periconceptional risk drinking, defined as 1 or more ounces of absolute alcohol (about two drinks) per day, was based on a patient’s recall of her alcohol intake during a 1-week period around the time of conception.

In Sokol and colleagues’ first study, the MAST; the CAGE test; and a tolerance question, “How many drinks does it take to make you feel high?” were evaluated in 971 subjects, 42 of whom were risk drinkers based on the 1-week recall. An analysis was conducted to determine which questions were useful in discriminating between risk and nonrisk drinkers. The analysis included the CAGE test and tolerance items only, and it revealed that all of these items contributed significantly to the prediction of risk drinking, except the question about having ever felt bad or guilty about drinking.

#### T-ACE

Based on these data, a new screening instrument, the T-ACE, was proposed. The Tolerance question scores two points if women need more than two drinks to get high; and the three CAGE test questions, Annoyed, Cut down, and Eye-opener, each score one point. Scores of two or more are considered positive evidence of risk drinking. Using the formulas outlined in table 1, T-ACE had a sensitivity of 76 percent in predicting periconceptional risk drinking compared with 59 and 76 percent for the CAGE test and the MAST, respectively. Specificities for the T-ACE and CAGE tests and the MAST were 79, 82, and 76 percent; positive predictive values were 14, 13, and 13 percent; and efficiency scores were 79, 80, and 76 percent. The T-ACE test was just as sensitive to risk drinking as the much longer MAST and was more sensitive than the CAGE test; differences in other measures of merit were relatively minor.

#### NET

In a refinement of the above work, items from the MAST, the CAGE test, and the T-ACE test that best identified risk drinkers were combined to form the NET questionnaire (Bottoms et al. 1989). The item taken from the MAST was, “Do you consider yourself a Normal drinker?” The item taken from the CAGE test was, “Do you ever have an Eyeopener?” The Tolerance question was taken from the T-ACE test; this item is counted positive if the answer given is more than two drinks. Any positive NET item is interpreted as a positive screening test. Evaluation was conducted in a sample of 2,042 (68 of whom reported risk drinking), using methods comparable with those described above. Sensitivity scores for the MAST, the CAGE test, the T-ACE test, and the NET test ranged from 50 to 54 percent, and specificity scores ranged from 94 to 98 percent. With sensitivity and specificity scores of 54 and 96 percent, respectively, the NET test performed somewhat better than did the other tests, and it was simpler. However, sensitivity was lower for all the tests than in the sample previously reported by Sokol and colleagues (1989).

Another version of the tolerance question was subsequently tested (Martier et al. 1990): “How many drinks can you hold?” This question is scored positive if women report being able to consume more than five drinks before falling asleep or passing out. When this version of the tolerance question was asked, the T-ACE test and the NET test each had a sensitivity of 91 percent and a specificity of 81 percent.

#### TWEAK

**Figure 1:**
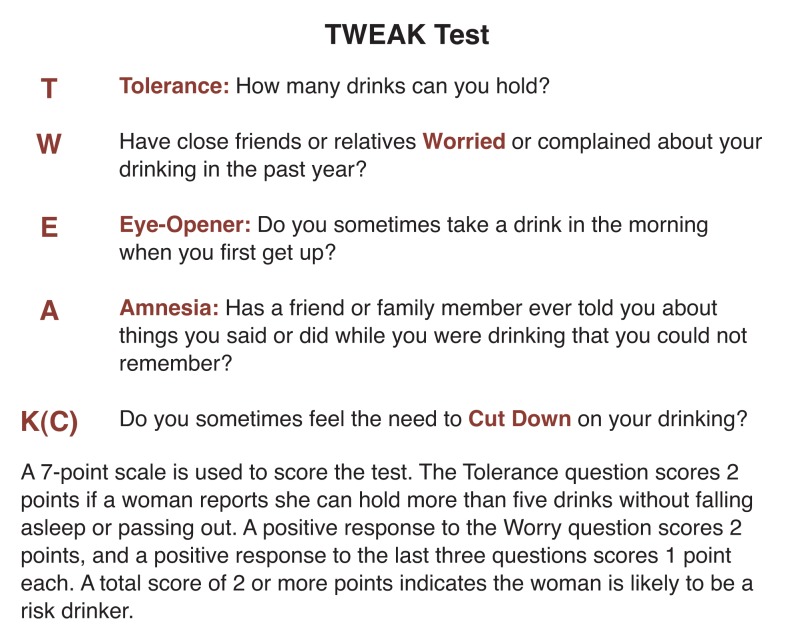
The TWEAK test is a screening tool for identifying women who are risk drinkers. It combines questions from the MAST, CAGE, and T-ACE tests that have been found most effective in identifying these women drinkers.

At the same time, Russell and Bigler (1979) had adapted an extended, self-administered form of the MAST for use in a female population. Questions were eliminated that asked about behavior that is more typical of male than female alcohol abuse, such as having fist fights after drinking. The researchers also eliminated questions about behavior that tends to identify individuals whose problems with alcohol have already been recognized and addressed, such as questions about attendance at Alcoholics Anonymous meetings. Studies of screening for alcohol abuse among obstetric patients revealed that three questions identified 70 percent of the women reporting two or more indications of problem drinking (Russell and Skinner 1988). The three questions concerned addressed blackouts, feeling the need to cut down on drinking, and having close friends or relatives worry or complain about the subjects’ drinking during the past year. These questions, plus the question on tolerance from the T-ACE test and a question on morning drinking from the CAGE test, were combined to form the TWEAK test (figure 1).

To score the TWEAK test, a seven-point scale is used. Positive responses to the tolerance and worry questions score two points each, and each of the last three questions scores one point for positive responses. A total score of two or more points indicates the woman is likely to be a risk drinker.

Sokol and colleagues recently compared the TWEAK test with the T-ACE test and the NET test for its ability to detect risk drinking, using the methods they established in their previous studies (S.S. Martier, personal communication, March 29, 1993). When the “how many drinks does it take to get you high” version of the tolerance question was asked, sensitivity scores for the TWEAK, T-ACE, and NET tests were 79, 70, and 64 percent, respectively, and specificity scores were 83, 85, and 86 percent. Sensitivity scores for the CAGE test and the MAST in the same population were only 49 percent, and specificity scores were 93 and 95 percent, respectively. When the “how much can you hold” version of the tolerance question was asked, sensitivity scores improved to 91, 89, and 87 percent for the TWEAK, T-ACE and NET tests, respectively, and specificity scores were 77, 79, and 80 percent. These findings are consistent with previous work indicating the superiority of the “hold” version of the tolerance question in identifying periconceptional risk drinking among black inner-city clinic patients in Detroit (Martier et al. 1990).

The TWEAK test appears to be somewhat more sensitive and less specific than the T-ACE or NET tests, but all three brief questionnaires clearly outperform the MAST and the CAGE test in screening for risk drinking during pregnancy. These evaluation studies need to be replicated in other regions of the country among obstetric populations representing other racial and socioeconomic backgrounds to establish the generalizability of these findings.

#### 4P’s

Another brief questionnaire currently under development for use in prenatal clinics is the 4P’s test (Burke and Caldwell in press). In this screen, four yes/no questions are asked about alcohol and/or drug use problems during a patient’s current Pregnancy, in her Past, in her Partner, and in her Parents. One positive answer to any question is considered an overall positive screen for a risk drinker. The 4P’s test is easy to administer and score, and questions about the past, partners, and parents may seem less threatening than questions about the patient’s current drinking behavior.

However, concerns have been raised about the test’s potential lack of specificity, the possibility that some patients would answer direct questions about alcohol or other drug use more readily than questions about problems related to such use, and whether questions on personal problems should be asked before less sensitive questions on partner and parent problems. Research to examine these issues and to validate the 4P’s test as a screening instrument is ongoing.

#### AUDIT

The brief questionnaires discussed so far have either asked about alcohol use directly or focused on consequences of alcohol use. An international group of alcohol researchers, who collaborated under the auspices of the World Health Organization, have combined both approaches in the Alcohol Use Disorders Identification Test (AUDIT; Saunders et al. 1993). The main screening instrument from this test is a 10-item questionnaire. Its purpose is the early identification of harmful drinking rather than alcohol disorders such as alcohol abuse or dependence.

Therefore, items were included that best distinguished light drinkers from harmful drinkers; however, the questionnaire also can detect alcohol disorders with a high degree of accuracy. The items selected tap three aspects of alcohol-related behavior: hazardous alcohol consumption, with three questions on alcohol intake (frequency, quantity, and frequency of six or more drinks at one time); dependence symptoms, with three questions on frequency; and harmful alcohol consumption, with four questions on problems caused by alcohol, including frequency of adverse psychological reactions. The timeframe used in the AUDIT is the past 12 months. Responses are multiple choice, with item scores ranging from 0 to 4 and totals ranging from 0 to 40; scores higher than 8 are considered positive.

Although the idea of asking both direct and indirect questions about risk drinking seems reasonable, and cross-national research demonstrated high sensitivity and specificity in identifying harmful/ hazardous drinkers, the AUDIT has not yet been evaluated in obstetric populations. Questions on alcohol intake precede those on consequences, and it is not clear whether asking about intake first would identify more risk drinkers or would trigger denial, reducing the number of risk drinkers identified. Denial is likely to be higher among obstetric patients than other types of patients, because they have probably been sensitized to the negative consequences of drinking during pregnancy (Nadler et al. 1987).

The AUDIT is longer and more complicated to score than some of the other brief questionnaires reviewed, and some of the items in the AUDIT overlap with other screening instruments (i.e., questions on blackouts, others worrying about the subject’s drinking, and morning drinking). Thus, further research is needed to determine whether the added items and more sophisticated scoring scheme make the AUDIT more sensitive to risk drinking during pregnancy than the TWEAK, T-ACE, or NET tests and whether the order of questions is optimal for detecting risk drinking.

The “true” status of patients regarding risk drinking is usually determined by asking them how much they drink. Because researchers depend on this type of intake question to assess the accuracy of questionnaires that ask about problems resulting from alcohol use, studies comparing the sensitivity and specificity of alcohol intake questions with alcohol-related problems questions have not been done. However, it is possible that some of the women who report alcohol problems but deny risk drinking are indeed risk drinkers and should be considered true, rather than false, positives on screening instruments that are based on questions about alcohol problems. Validity studies that more accurately determine risk drinking during pregnancy are needed to investigate this possibility.

## Clinical Applications and Implications

Health care providers consulted by women in their childbearing years could incorporate into their practices brief questionnaires to screen for risk drinking. Self-administered questionnaires on alcohol intake or consequences of drinking could be included with questions on health that patients answer when they first enter care.

Brief questionnaires to screen for prenatal behavioral risk factors, including alcohol and other drug use, also have been successfully administered via interactive computer programs (Lapham et al. 1991). Such programs flash questions about risky behavior on a computer screen, and women can indicate their answers by keying in their responses. Most programs produce a report summarizing risky behaviors in need of modification for health care providers. Some evidence suggests that people are more likely to report indications of risk drinking on self-administered questionnaires or computer interviews than to physicians (Russell and Bigler 1979; Lapham et al. 1991). Also, physicians may be reluctant to initiate a discussion of alcohol use with their patients, perhaps fearing that patients will become annoyed and forgo needed prenatal care (Russell et al. 1983).

Although this review focuses on the assessment of risk drinking during pregnancy, alcohol-related birth defects have been associated with drinking during early pregnancy, perhaps even before women realize that they are pregnant (Russell and Skinner 1988). Accordingly, the most effective intervention to prevent alcohol-related birth defects would reduce heavy drinking prior to conception. However, as the central nervous system of the fetus develops throughout pregnancy, intervention at any point during pregnancy has the potential to minimize mental retardation and cognitive developmental deficits, the most serious long-term consequences of prenatal alcohol exposure.

The effectiveness of intervention to reduce risk drinking during pregnancy is enhanced by physiological factors associated with pregnancy that reduce women’s desire to drink, by their desire for a healthy child, and by support from family members and medical providers.

It is crucial that efforts made to limit risk drinking during pregnancy be maintained after the baby is born. There is a tendency for women who substantially reduce their alcohol intake during pregnancy to resume heavy drinking post partem, thus putting at risk their own health and subsequent pregnancies. This tendency may be particularly strong among women who have previously given birth to an alcohol-affected child. Indeed, the best predictor of alcohol-related birth defects is having an older sibling with FAS or FAE. A woman’s return to heavy drinking may be exacerbated by her guilt and by the stress of caring for a child with developmental deficits.

## Summary

Assessing risk drinking during pregnancy is an essential step toward educating pregnant women about the potential dangers of such behaviors. The utility of using brief questionnaires, the TWEAK, T-ACE, and NET tests, to screen for periconceptional risk drinking has been demonstrated in pregnant black women attending an inner-city clinic in Detroit. A strength of these questionnaires is the inclusion of questions on tolerance that indirectly assess risk drinking during pregnancy. In this population, asking how much alcohol a woman can consume before she falls asleep or passes out appears to be more sensitive to risk drinking than asking how much it takes to make the woman feel high. In addition, these questionnaires employ questions on consequences of heavy alcohol use, such as friends and family expressing concern about one’s drinking, which often occur early in the development of alcohol disorders.

Direct questions on alcohol intake can potentially identify risk drinkers who may not have experienced negative consequences of alcohol use. However, studies designed to verify the validity of self-reported alcohol intake during pregnancy are needed to evaluate the influence of denial. Additional research on assessing risk drinking during pregnancy is needed to evaluate new screening instruments such as the AUDIT and the TWEAK test in obstetric patients who have a wide range of sociodemographic characteristics.

## Figures and Tables

**Table 1:** Evaluation of a Screening Test in a Population Having a 5-Percent Prevalence Rate of Risk Drinking

	True Risk Drinking Status		
		
	Positive	Negative	Screening Test Totals

Results of Screening Test			

Positive	a (4)	b (10)	a + b (14)

Negative	c (1)	d (85)	c + d (86)

True Status Total	a + c (5)	b + d (95)	(100)

a = The number of women for whom the screening test for risk drinking is positive, and they actually are risk drinkers (true positive).			
b = The number of women for whom the screening test for risk drinking is positive, but they are not risk drinkers (false positive).			
c = The number of women for whom the screening test for risk drinking is negative, but they are risk drinkers (false negative).			
d = The number of women for whom the screening test for risk drinking is negative, and they are not risk drinkers (true negative).			
$\begin{array}{l} {\text{Sensitivity}}^{1}=\frac{\text{a}}{\text{a}+\text{c}}=\frac{4}{5}=80.0\%\\ {\text{Specificity}}^{2}=\frac{\text{d}}{\text{b}+\text{d}}=\frac{85}{95}=89.5\%\\ \text{Positive Predictive }{\text{Value}}^{3}=\frac{\text{a}}{\text{a}+\text{b}}=\frac{4}{14}=28.6\%\\ {\text{Efficiency}}^{4}=\frac{\text{a}+\text{d}}{\text{a}+\text{b}+\text{c}+\text{d}}=\frac{89}{100}=89.0\% \end{array}$			

1 The probability that a risk drinker is positive on the test.

2 The probability that a nonrisk drinker is negative on the test.

3 The probability that a woman with a positive screening score is a risk drinker.

4 The overall percentage of women correctly identified.

## Footnotes

1 The terms “alcohol abuse” and “dependence” are used as defined by the American Psychiatric Association’s *Diagnostic and Statistical Manual of Mental Disorders, Third Edition, Revised*, 1987.

2 The term “alcoholism” as it is used in this article does not denote a specifically defined disorder.
